# Identification and Prioritization of Important Attributes of Disease-Modifying Drugs in Decision Making among Patients with Multiple Sclerosis: A Nominal Group Technique and Best-Worst Scaling

**DOI:** 10.1371/journal.pone.0164862

**Published:** 2016-11-03

**Authors:** Ingrid E. H. Kremer, Silvia M. A. A. Evers, Peter J. Jongen, Trudy van der Weijden, Ilona van de Kolk, Mickaël Hiligsmann

**Affiliations:** 1 Department of Health Services Research, CAPHRI School of Public Health and Primary Care, Maastricht University, Maastricht, The Netherlands; 2 Public Mental Health, Trimbos Institute, Netherlands Institute of Mental Health and Addiction, Utrecht, The Netherlands; 3 MS4 Research Institute, Nijmegen, The Netherlands; 4 Department of Community & Occupational Medicine, University Medical Centre Groningen, Groningen, The Netherlands; 5 Department of Family Medicine, CAPHRI School of Public Health and Primary Care, Maastricht University, Maastricht, The Netherlands; 6 Department of Health Promotion, NUTRIM School of Nutrition and Translational Research in Metabolism, Maastricht University, Maastricht, The Netherlands; Heinrich-Heine-Universitat Dusseldorf, GERMANY

## Abstract

**Objectives:**

Understanding the preferences of patients with multiple sclerosis (MS) for disease-modifying drugs and involving these patients in clinical decision making can improve the concordance between medical decisions and patient values and may, subsequently, improve adherence to disease-modifying drugs. This study aims first to identify which characteristics–or attributes–of disease-modifying drugs influence patients´ decisions about these treatments and second to quantify the attributes’ relative importance among patients.

**Methods:**

First, three focus groups of relapsing-remitting MS patients were formed to compile a preliminary list of attributes using a nominal group technique. Based on this qualitative research, a survey with several choice tasks (best-worst scaling) was developed to prioritize attributes, asking a larger patient group to choose the most and least important attributes. The attributes’ mean relative importance scores (RIS) were calculated.

**Results:**

Nineteen patients reported 34 attributes during the focus groups and 185 patients evaluated the importance of the attributes in the survey. The effect on disease progression received the highest RIS (RIS = 9.64, 95% confidence interval: [9.48–9.81]), followed by quality of life (RIS = 9.21 [9.00–9.42]), relapse rate (RIS = 7.76 [7.39–8.13]), severity of side effects (RIS = 7.63 [7.33–7.94]) and relapse severity (RIS = 7.39 [7.06–7.73]). Subgroup analyses showed heterogeneity in preference of patients. For example, side effect-related attributes were statistically more important for patients who had no experience in using disease-modifying drugs compared to experienced patients (p < .001).

**Conclusions:**

This study shows that, on average, patients valued effectiveness and unwanted effects as most important. Clinicians should be aware of the average preferences but also that attributes of disease-modifying drugs are valued differently by different patients. Person-centred clinical decision making would be needed and requires eliciting individual preferences.

## Introduction

Multiple sclerosis (MS) is a demyelinating and degenerative disease of the central nervous system causing physical and cognitive disabilities. MS occurs as different disease courses [1]. Relapsing-remitting MS (RRMS) is characterized by recurring exacerbations of MS symptoms (relapses) that recover partially or completely (remission) [1]. Between relapses, the disease remains stable. Progressive types of MS are characterized by a continuous increase in disability over time, either from the onset of MS (primary progressive MS) or conversion of RRMS to secondary progressive MS [1]. When a central nervous system demyelinating event has occurred that is isolated in time and compatible with the possible future development of MS, clinically isolated syndrome (CIS) is diagnosed [2]. Thirteen different disease-modifying drugs (DMDs) are currently available in the United States and in Europe to reduce the relapse rate and disease progression for patients with RRMS and new DMDs are still being developed. Some of these DMDs are also indicated for the treatment of CIS [3–5]. Adherence to DMD treatment is problematic, however, ranging from 41% to 88% of doses taken as prescribed [6], and non-adherence is associated with an increased relapse rate [7].

DMDs differ in their effectiveness, unwanted effects and other characteristics or attributes [3, 5]. For patients diagnosed with RRMS or CIS, a decision needs to be made between the options the patient has, including the option of no DMD treatment. Decision making can be difficult because it requires comparing different DMD treatment options according to their specific characteristics or attributes.

Patients with MS have been reported to prefer being actively involved in the decision making about DMDs [8]. Therefore, it is important to inform and involve patients in the decision to start or not start taking a DMD, and, in case of starting, it is important to choose the type of DMD that best suits the patient’s preferences and situation. In the shared decision making approach, the decision is made through a joint process between the physician and the patient. This entails informing the patient about treatment options and deliberation with the physician about which treatment would best fit the patient’s preferences [9]. Understanding which DMD attributes are important according to patients may therefore contribute to the tailoring of information for patients in clinical practice and may support the clinical decision making process. Effective support of the shared decision making process could improve patient satisfaction and treatment adherence [10].

Preference research is often used to elicit patients’ preferences for treatment options, i.e. to determine which attributes of the treatment options are important for patients in decision making. Patients are asked to state which treatment or treatment attribute they prefer in hypothetical trade-offs between two or more treatments or attributes [11]. Some preference studies have been conducted on MS patients’ preferences for DMD treatments [12–18] but, to the best of our knowledge, no study has attempted to identify among patients the full range of attributes of DMDs in general–regardless of the specific type or administration mode of the DMD–that may be of importance in decision-making. Therefore, important attributes of DMDs for decision making between all available DMDs may have been omitted in the exercises for prioritization of the attributes.

The current study aimed to use thorough research methodologies for evaluating patients’ preferences for the full spectrum of DMD attributes that are of importance in the decision about DMD treatment. More specifically, the study’s objective was twofold. The first objective was to identify the range of DMD attributes that influence the decision from the patients’ perspective. The second objective was to quantify the relative importance of the identified attributes among a large group of patients.

## Methods

**Fig 1:**
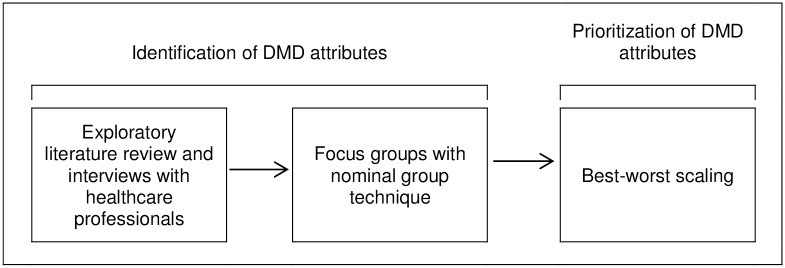
Consecutive process of studies.

Consecutive studies were conducted (Fig 1). First, an exploratory literature review and telephone interviews with healthcare professionals were performed to identify DMD attributes that may be of importance for decision making in DMD treatment. A full description of the methods used and results of this exploratory phase is provided in S1 and S2 Texts. Next, patient focus groups using a nominal group technique were formed to identify attributes and to verify any additional attributes that were derived from the exploratory phase. A nominal group technique, as developed by Delbecq and Van de Ven [19, 20], is a structured method for guiding a group discussion to generate and prioritize ideas for a specific question. The RATS guideline [21] was used for reporting the methods and results of the nominal group technique, when appropriate. Finally, a best-worst scaling was conducted to prioritize the attributes according to a patient sample much larger than the number of patients that participated in the focus groups. A best-worst scaling is a specific method for conducting preference research. Respondents are asked to complete a series of choice tasks in which they have to choose the most and least important attributes from a selection of 4 or 5 attributes from a master list of attributes [22]. The advantage of the best-worst scaling over other stated preference research is the ability to acquire patients’ preferences for a large number of attributes, regardless of the levels of the attributes. As the best-worst scaling was administered online, design and results of the best-worst scaling were presented according to the Checklist for Reporting Results of Internet E-Surveys [23]. The protocols of both the focus groups and the best-worst scaling were submitted to the Medical Ethics Committee of the Academic Hospital Maastricht and Maastricht University, the Netherlands (nr. 14-4-172). The committee concluded that the study did not qualify for a review according to the Dutch Medical Research Involving Human Subjects Act of 1998 and provided a positive decision for conducting the study. The study was performed in agreement with the ethical standards laid down in the 1964 Declaration of Helsinki and its later amendments. All participants gave their written informed consent before participating in the studies.

### Patient population

Both for the focus groups with nominal group technique and the survey with the best-worst scaling, patients were considered eligible for participation if they were diagnosed with RRMS or CIS, were 18 years or older, and were willing and able to participate. For the focus groups an additional criterion was that the participant had experience with making a decision about DMD treatment or had experience with taking DMDs. Potential participants were recruited through advertisements on websites, social media or mailing lists of MS patient organizations.

Patients interested in participating in the focus groups were asked to contact one of the researchers (I.K.) by phone or e-mail. Based on the respondents’ place of residence, three locations dispersed over the Netherlands were selected: a hospital in Nijmegen (the eastern part of the Netherlands) and community centers in Nieuwegein (the middle of the Netherlands) and Roermond (the southern part of the Netherlands). Patients who fulfilled the inclusion criteria and were able to travel to one of the selected locations were sent an information leaflet about the study and a consent form by mail. After a week, the patients were contacted by the first author to answer any of their questions and to register their participation. After participation in the focus group, each respondent received a 50 Euro gift card to compensate for any travel expenses and the time invested. Patients willing to participate in the best-worst scaling were redirected to the online questionnaire by means of a link in the advertisement or in the e-mail. After providing information about the purpose and content of the survey, the patients were informed that by filling out the questionnaire, they gave consent to the use of their answers in the study. To prevent the same person filling out multiple questionnaires, cookies were placed on their browser when they submitted their questionnaire. The recruitment method did not allow us to identify which patients did not choose to participate in the best-worst scaling or the focus groups.

### Focus group with nominal group technique

#### Design

Focus groups were performed by applying the nominal group technique. The structured method of a nominal group technique ensures that every participant’s perspective is included and allows for differences in perspectives [19, 20]. A nominal group technique is therefore particularly suitable for identifying the full range of important DMD attributes and has already been used in other studies, e.g. to identify attributes of osteoporotic medications [24]. Two researchers were present at each focus group to facilitate the group discussion (IK) and to take notes (IvdK). The nominal group technique consisted of four steps. First, the participants were asked to individually answer the following question: “*What characteristics of DMDs do you feel are important to consider when having to make a decision about DMD treatment*?*”* Second, the participants took turns in reporting attributes until all attributes generated were written down on a flip-over by the facilitator. This ensured that every participant’s opinion was elicited, and that they participated in the discussion. Third, the discussion was intended to come to an agreement within the group about the meaning and scope of each attribute. The participant that reported the attribute often provided the first description. Other participants were given the opportunity to react to this description. If the descriptions were different or had a broad scope an attribute was split into multiple attributes. If multiple attributes were similar in their meaning, these attributes were combined. This was done upon agreement of the participants. In the second and third focus group, the discussion was followed by asking for the participants’ opinions about any additional attributes derived from the exploratory phase and whether these attributes should be included. Participants were entirely free to accept or reject the additional attributes. In the final step of each nominal group technique, participants were asked to select the 10 most important attributes from the list of attributes compiled and rank the top 5. After the third focus group, it was checked whether data saturation had been reached, i.e. whether any new attributes emerged that had not already been derived from the previous focus groups or the exploratory phase. Responses during the nominal group technique were recorded on audio tape so that attribute descriptions could be transcribed correctly and these tapes were erased afterwards. The anonymity of the respondents was ensured in the transcriptions.

#### Analyses

An overall list of important DMD attributes for decision making according to patients was created by comparing the attribute definitions from the three groups to each other. If from the transcripts of the discussions it appeared that attributes had similar descriptions across the focus groups, then these attributes were combined. The frequency with which participants included the attributes in their top 10 and top 5 was calculated. The attributes in the top 5 were awarded points, from 5 points for the most important attribute to 1 point for the least important one. Per attribute, the mean importance score was calculated by dividing the total points awarded per attribute by the total number of patients participating in all focus groups. Based on the mean importance score and calculated frequencies, an initial ranking of attributes was made from most to least important. Attributes that were not included in any of the participants’ top 10 of most important attributes were excluded from the best-worst scaling.

### Best-worst scaling

#### Design

**Fig 2:**
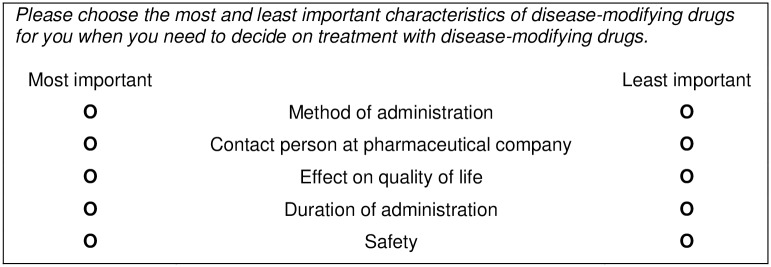
Example of a choice task in the best-worst scaling.

Based on the results of the focus groups, a best-worst scaling was developed and converted to an online questionnaire of 3 pages with 6 questions each, and also contained 2 pages with 4 or 5 questions about the respondent’s demographic and disease characteristics. All questions had to be filled out before the respondents could proceed to the next page or submit the questionnaire, but respondents were able to go back to change their answers before submission. A “don’t know” option was provided for the appropriate demographic questions. Responses were registered automatically. No data were collected that could be referred back to the identity of the respondent (e.g. IP-address). The best-worst scaling consisted of 17 unique choice tasks. Each choice task presented five attributes of the full attribute list as derived from the focus groups. Each respondent was asked to select the most and least important attributes for decision making about DMD treatment. The attributes selected by the respondent represent the attributes that are furthest apart on the importance scale for the individual patient [22]. Additionally, patients were provided the opportunity to list any important DMD attributes that in their opinion were not included in the best-worst scaling. Fig 2 provides an example of a choice task.

A fractional design was created for the best-worst scaling with Sawtooth SSI Web version 8.2.0. This software creates the most efficient design, characterized by orthogonality (the frequency of an attribute paired with other attributes is equal for all attributes), balance (the frequency of attributes occurring in the best-worst scaling is equal), and positional frequency (the frequency of attributes on the 1^st^ to 5^th^ position in the choice task is equal) and determines which attributes are presented to the respondent in each choice task. Four best-worst scaling versions were created. Each attribute was presented 12 or 13 times, was combined at least once with every other attribute and appeared 2 to 4 times in each position in the choice tasks. Respondents randomly received 1 of the 4 best-worst scaling versions. The questionnaire was pilot-tested among researchers (N = 3) and MS patients (N = 3) prior to the start of the study, which resulted in minor revisions of the instructions. The questionnaire was not found to be too cognitively burdensome to patients.

#### Analyses

Questionnaires were filled out from 12 May 2015 to 5 June 2015. Only completed best-worst scaling questionnaires were included in the analyses. Descriptive statistics were used to present demographic and disease characteristics of the respondents. Hierarchical Bayes analysis was performed with Sawtooth SSI Web version 8.2.0 to estimate the mean relative importance score per attribute. The raw score, which was obtained with an iterative process of estimating individual utility scores based on the sample means, was rescaled to a probability score on a ratio scale. This score represents the attribute’s relative importance for decision making about DMD treatment according to the respondent. The relative importance scores of all attributes combined for an individual respondent sum up to 100 [25]. A mean overall relative importance score was calculated per attribute with its 95% confidence interval. Based on the mean scores, attributes were ranked from most to least important for decision making in DMD treatment. Attributes with a score of 3.7 were regarded as of average importance (100 points divided by 27 attributes). In lack of consensus on the minimal important difference in relative importance scores, if confidence intervals of two consecutive ranked attributes did not overlap, we considered them to be of different importance in the decision about DMDs.

The quality of the responses was checked based on the individual’s fit statistic, i.e. if responses had a fit statistic below 0.247, these were excluded from analyses because this indicates purely random responses to the choice tasks [26]. Subgroup analyses on gender, age, education, disease duration, relapse rate, experience with DMD, and current and prior DMDs taken were conducted to explore whether patients’ preferences for DMD attributes differ according to demographic characteristics, disease characteristics or DMD experience. Subgroups for continuous data (age, disease duration) were made according to the median. For categorical data, subgroups were made based on relevance for comparison (e.g. DMD-naive vs. DMD-experienced). Difference in importance scores between subgroups were statistically tested with an independent t-test for parametric data and the Mann-Whitney test for non-parametric data using SPSS for Windows version 20. An alpha of .05 and a Bonferroni-adjusted alpha of .0019 (for 27 comparisons) were used to assess whether differences in RIS of attributes between subgroups were statistically significant.

## Results

### Patient population

Table 1 presents the characteristics of the patients that participated in the focus groups and the best-worst scaling. Three focus groups with a total of 19 RRMS patients took place. Each group consisted of male and female participants ranging in age, educational level and experience with DMD use. Age was approximately normally distributed with a mean of 46.8 (±8.8) years old. Thirteen (68.4%) patients had prior experience with making a decision about DMDs and six (31.6%) were considering or reconsidering their DMD options at the time of the focus groups.

**Table 1:** Patient characteristics: nominal group technique (N = 19) and best-worst scaling (N = 185).

Characteristics		Nominal group technique	Best-worst scaling
**Women n (%)**		15 (78.9)	160 (86.5)
**Age, mean ± SD (years)**		46.8 ± 8.8	42.1 ± 9.6
**Educational level n (%)**			
	**Lower**	7 (36.8)	72 (38.9)
	**Higher**	12 (63.2)	113 (61.1)
**Employed**			
	**Yes**	8 (42.1%)	90 (48.6%)
	**No**	11 (57.9%)	95 (51.4%)
**Diagnosis**			
	**RRMS n (%)**	19 (100)	182 (98.4)
	**CIS n (%)**	0 (0)	3 (1.6)
**Duration of diagnosis, mean ± SD (years)**		9.5 ± 8.4	6.4 ± 5.9
**Relapse rate previous year, n (%)**		(N = 17)	(N = 172)
	**0**	7 (41.2)	63 (36.6)
	**≥1**	10 (58.8)	109 (63.4)
**Currently taking DMD n (%)**		15 (78.9)	131 (70.8)
**Previously taken DMD n (%)**		10 (52.6)	86 (46.5)
**Number of prior taken DMD n (%)**			(N = 184)
	**1**	6 (31.6)	43 (23.4)
	**2**	2 (10.5)	35 (19.0)
	**3**	2 (10.5)	5 (2.7)
	**4**	0 (0)	3 (1.6)

CIS, clinically isolated syndrome; DMD, disease-modifying drug; RRMS, relapsing-remitting multiple sclerosis; SD, standard deviation.

Of the 286 people who accessed the survey via the provided link, 193 respondents (67.5%) started the best-worst scaling exercise and met the inclusion criteria. Of these respondents, 185 people (95.9%) completed the best-worst scaling exercise. The majority of the respondents who completed the best-worst scaling exercise were female (86.5%); this is somewhat higher than the Dutch MS population of 72% [27]. Of the respondents, 54.1% had completed a higher vocational education or university education. Mean age was 42.1 (±9.6) years, which was approximately normally distributed, and almost all patients reported a diagnosis of RRMS (98.4%). Twenty-seven (14.6%) patients reported having no experience with using DMDs and 131 (70.8%) respondents were currently taking a DMD.

### Focus group with nominal group technique

Data saturation was reached in the third focus group because no new attributes emerged. Combining the attribute lists from the three focus groups resulted in 34 DMD attributes that, according to the patients, were important in decision making about DMD treatment. Based on the mean importance scores, participants in the focus groups regarded the type of side effects as most important, followed by effect on disease progression, method of administration, effect on relapse rate, safety and insurance coverage. Seven attributes were not included in the top 10 by any of the participants (adherence rate, availability of the DMD in the Netherlands, brand recognition, issuance of DMD, wash-out requirements, shelf life and legal liability). For descriptions of all DMD attributes, their ranking and mean importance scores, we refer to the data in S3 Text.

### Best-worst scaling

The best-worst scaling survey included 27 attributes. From the list of 34 attributes identified in the focus groups, seven attributes were excluded because they were not included in the top 10 by any of the participants in the focus groups. All respondents had a fit statistic higher than 0.247 and were therefore all included in the analysis. Hierarchical Bayes analysis showed that attributes related to effectiveness and unwanted effects were most important for decision making according to patients. Table 2 presents the group average relative importance scores of all 27 attributes with their 95% confidence intervals ranked from most to least important for decision making in DMD treatment. The effect on disease progression was ranked as the most important attribute with a RIS of 9.6 (95% CI 9.5–9.8). The effect on quality of life, defined as the overall increase in the well-being of a patient as a result of the DMD, was ranked second (RIS 9.2; 95% CI 9.0–9.4), followed by effect on the relapse rate (RIS 7.8; 95% CI 7.4–8.1), and severity of side effects (RIS 7.6; 95% CI 7.3–7.9). The severity of side effects scored higher than safety issues, i.e. common side effects were found to be of more influence on the treatment decision than risks of life threatening or severely disabling consequences. The most important attribute not related to beneficial or unwanted effects was influence on life style (RIS 5.3; 95% CI 4.9–5.7) but this attribute was only half as important as the number one ranked attribute, effect on disease progression. Other convenience issues with taking DMDs were valued far less: the RIS for administration method, administration frequency and required monitoring were 1.6 (95% CI 1.2–2.0), 0.7 (95% CI 0.5–0.9) and 0.6 (95% CI 0.4–0.7) respectively. Rankings of attributes as derived from the focus groups deviate from these results. The convenience issues mentioned previously were all ranked in the top 10, while the effect on quality of life, plaque development and severity of side effects were of less importance (Online Resource 3).

**Table 2:** Group average relative importance scores of attributes in decision making as derived from the best-worst scaling among 185 patients.

Attribute	RIS	95% CI lower bound	95% CI upper bound
**Effect on disease progression**	9.64	9.48	9.81
**Effect on quality of life**	9.21	9.00	9.42
**Effect on relapse rate**	7.76	7.39	8.13
**Severity of side effects**	7.63	7.33	7.94
**Effect on the severity of relapses**	7.39	7.06	7.73
**Effect on current MS symptoms**	7.32	7.03	7.60
**Effect on plaque development in the brain**	7.31	6.94	7.67
**Safety**	6.04	5.62	6.47
**Influence on lifestyle**	5.31	4.88	5.73
**Type of side effects**	5.00	4.60	5.39
**Effect on life expectancy**	4.81	4.36	5.27
**Uncertainty about long-term consequences**	4.58	4.18	4.98
**Duration of side effects**	3.74	3.45	4.02
**Pace of effect**	3.18	2.87	3.50
**Insurance coverage**	2.71	2.29	3.12
**Interaction with other medication**	1.72	1.46	1.99
**Method of administration**	1.58	1.19	1.97
**Mode of action of DMD**	0.99	0.81	1.17
**Further development of DMD**	0.87	0.73	1.00
**Total DMD costs**	0.86	0.68	1.04
**Frequency of administration**	0.68	0.48	0.88
**Required monitoring**	0.55	0.38	0.72
**Use of DMD among other MS patients**	0.34	0.26	0.42
**Ease of travelling**	0.29	0.17	0.42
**Duration of administration**	0.20	0.17	0.24
**Composition of DMD**	0.18	0.13	0.23
**Contact person at pharmaceutical company**	0.10	0.06	0.14

CI, confidence interval; DMD, disease-modifying drug; RIS, relative importance score.

One MS patient reported “effect of the DMD on pregnancy or child” as an additional attribute that was not already included in the best-worst scaling. Other attributes that were reported, such as effect of the DMD on mental well-being and family members, and feelings of depression or anxiety, were captured in or had considerable overlap with other attributes.

**Fig 3:**
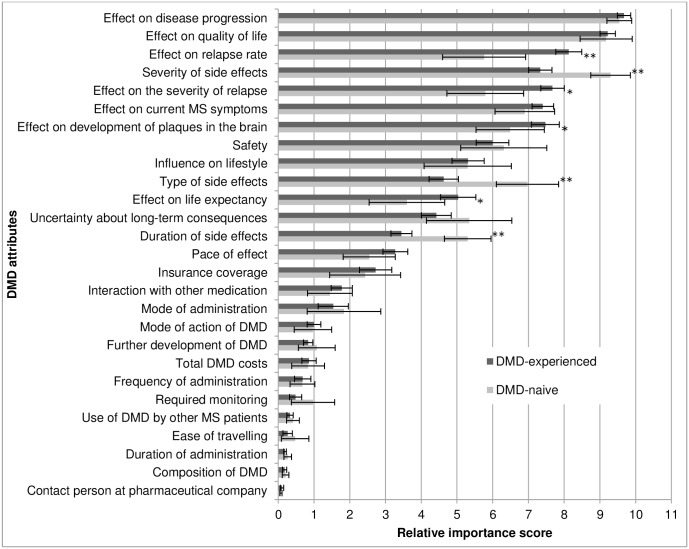
Attributes’ relative importance score: DMD-naive (n = 27) vs. DMD-experienced patients (n = 157). Attributes are ordered according to ranking of the overall analysis. The x-axis indicates the relative importance score per attribute with its 95% confidence interval. * p < .05, ** p < .0019.

Subgroup analyses showed deviations from the overall importance scores for patients with certain characteristics. As is presented in Fig 3, patients who had never used a DMD (n = 27) valued duration, type and severity of side effects significantly higher than did patients who had DMD experience (n = 157) (respectively U = 946, z = -4.59, p < .001; t(182) = 4.36, p < .001; U = 863, z = -4.92, p < .001). The patients in this last group had higher relative importance scores for the effectiveness of the DMD on the relapse rate, relapse severity, plaque development and life expectancy compared with DMD-naive patients (respectively U = 1196, z = -3.61, p < .001; U = 1312, z = -3.16, p = .001; U = 1585, z = -2.09, p = .038; U = 1549, z = -2.23, p = .026). For attributes of which the relative importance score did not significantly differ between DMD-naive and DMD-experienced patients, the results of the analyses are provided in S1 Table.

Similar differences in the importance of attributes were found for patients who were not using a DMD at the time of survey administration (n = 54) in comparison with patients using a DMD (n = 131): attributes concerning effectiveness were more important in the decision for patients currently using a DMD, while patients who were not using a DMD valued attributes related to unwanted effects significantly higher. Although ranked only 16^th^, the administration method was more important for patients taking orally administered DMDs (mean RIS = 2.5; 95% CI 1.6, 3.4) in comparison with patients taking parenteral (intramuscular, subcutaneous or intravenous) DMDs (mean RIS = 1.0; 95% CI 0.6, 1.5), which was a significant difference (U = 1416, z = -3.28, p = .001). The safety of the DMD was significantly more important for patients with a diagnosis of MS longer than 4.6 years compared to patients with shorter disease durations (U = 3174, z = -3.03, p = .001). Male patients were significantly more concerned about the influence of DMD use on lifestyle and with the effect on life expectancy in comparison with female patients (U = 1366, z = -2.55, p = .011; U = 1468, z = -2.14, p = .032). No significant differences were found in the importance scores of attributes of higher and lower educated patients. Results of all subgroup analyses are provided in S1 Fig.

## Discussion

The current study aimed to identify the full spectrum of DMD attributes and to quantify their relative importance in decision-making about DMD treatment according to RRMS and CIS patients. Patients reported a total of 34 different attributes that might influence their decision. Quantification of the relative importance showed that, as a group, patients place the most emphasis on benefits–especially disease progression and quality of life–and on unwanted effects when having to make a decision in DMD treatment, rather than usability issues. The ranking of the attributes showed that the most important attributes are comparable to the attributes used in other stated preference research [12–18]. Preventing disease progression was found in previous preference research to be an important attribute of DMDs [12–18] but quality of life has not yet been reported in these studies. The effect on quality of life–the increase or decrease in the overall well-being of a patient as a result of the DMD–could be interpreted as a summarizing attribute, incorporating the DMD’s beneficial effects and burdens into one attribute. This could explain its importance for patients. However, data from high quality randomized controlled trials on the effects of DMDs on quality of life are lacking for DMDs that have been available for the treatment of MS for some time now. It is encouraging that new RCTs and many observational studies are including quality of life as an outcome measure, since the effect of the DMD on quality of life is regarded as important information by the patients.

Regarding unwanted effects, the severity of non-life threatening physical and psychological side effects–essentially, the extent to which these side effects outweigh the desire to treat MS–was found to be the most influential attribute in decision making. This is in contrast to the results from the most comprehensive study about preferences for DMD attributes conducted among a large number of RRMS patients, which found life-threatening or severely disabling side effects to be the most important attribute and minor side effects to be the least important [17]. However, this study included four levels for life-threatening side effects denoted as risk of death or becoming severely disabled (0 out of 1,000; 0.5 out of 1,000; 1 out of 1,000; or 10 out of 1,000), while common side effects were split into three levels of types of common side effects (headaches and muscle or joint aches; increased risks of infection; and mood changes). Our study did not include levels, and therefore these could not influence how patients valued the attributes, perhaps explaining the differences between the two studies’ findings.

Noteworthy are the relatively low ranking and importance scores of administration method and administration frequency, ranked 17^th^ and 21^st^ respectively, as derived from the best-worst scaling, while in the literature these attributes were found to be of substantial importance in decision making among MS patients [17, 28, 29]. Attributes that were valued more highly than mode and frequency of administration by patients in our study, such as insurance coverage, total costs, continuous development of the DMD and interaction with other medication, were not included in other studies. Moreover, influence on lifestyle was valued relatively highly in our study. Although it was described to patients as “the extent to which a patient’s habits or lifestyle have to be adjusted for proper use of the medication, such as the flexibility in time of administration, restrictions on consuming alcohol, driving, sports, work, etc.”, patients may have also included administration method and frequency in this attribute, resulting in a higher ranking in comparison with administration method and frequency.

Subgroup analyses showed heterogeneity in preferences of patients according to different characteristics. For example, attributes related to unwanted effects were stated to be more influential in decision making by patients with no prior DMD experience in comparison with patients who had experience with DMD use. These findings reinforce the individuality of preferences and the need to incorporate the individual’s perspective into the clinical decision making process. It underlines the need for shared decision making, as this approach is focused on supporting patients in developing informed preferences based on objective information [30].

By investigating patients’ preferences about DMD attributes, our study may help to identify which information patients need about DMDs in order to make an informed decision, therefore enabling clinicians to adjust their information provision accordingly to facilitate the process of shared decision making and to support the development of informed preferences in patients. However, clinicians should also take into account that the study results provide guidance for the average patient. As heterogeneity in the results show, different patients could find different attributes of DMDs important for decision making. Therefore, clinicians should inform each patient who wants to be involved in the decision making about the pros and cons per option based on the patient’s personal preferences. As a result, clinicians should support the patient to elicit what is important to him or her and adjust the consultation accordingly, i.e. incorporate the patient’s preference in the decision or delay decision making and empower the patient with a patient decision aid. A patient decision aid contains concise summaries of the evidence on important attributes of the treatment options and includes a preference clarification method to assess what attributes of the treatment options are of importance for the individual patient. A decision aid could therefore support the shared decision making process in clinical practice [31]. The results of the current study will inform the development of such a decision aid for MS patients who need to make a decision about DMD treatment.

A strength of our study is the thorough methodology of a nominal group technique followed by a best-worst scaling used for identification and prioritization of attributes. Using focus groups with a nominal group technique ensured the direct elicitation of patients’ perspectives on all important DMD attributes for decision making, whereas prior preference studies focused only on a selection of attributes and/or relied on literature review and consultations with clinical experts for identification of attributes. Comparing attribute rankings from the nominal group technique and the best-worst scaling, there were considerable differences. Although data saturation was achieved in the identification of DMD attributes in the nominal group technique, we did not aim for data saturation in the ranking of the attributes. Due to variation in the attributes reported per nominal group technique, not all 34 attributes were included in the rankings of each focus group, resulting in lower validity of the overall ranking in the nominal group technique. In addition, the ranking in the nominal group technique was established after an interactive process of participants discussing the attributes and possibly influencing each other’s opinion in comparison to the best-worst scaling in which respondents filled out the survey by themselves. The differences in rankings emphasize the importance of using a quantitative method such as a best-worst scaling for prioritization of the attributes, in addition to qualitative work on identifying attributes.

Some limitations should be taken into account with the interpretation of the results. We used patient-centered methodologies for identifying the attributes. However, we cannot exclude that attributes important for some patients were not included in the study. In the best-worst scaling, one patient reported an additional attribute to be important: the effect of the DMD during pregnancy on the unborn child. Although it is generally recommended to discontinue DMD treatment before trying to conceive, treatment with certain DMDs can be continued in case of highly active MS [32–36]. Moreover, during DMD treatment patients may become pregnant inadvertently. A possible reason for omission of this attribute in the best-worst scaling may be that the 19 patients questioned in the focus groups and the six healthcare professionals questioned by interview regarded these risks inherent to safety or side effects. Another reason for omission by the patients in the focus groups may be that they already had children or stopped thinking about having children as the average age of patients in the focus groups was 46.8 years (SD 8.8), meaning that 68% of patients were between 38.0 and 55.6 years old and ranging from 30 to 68 years old. Actually, only 2 out of 15 women in the focus groups were between 30 and 35 years of age, the other being older. Thus, the number of women in child-bearing age was relatively low, which could explain the omission of the attribute regarding pregnancy risks. Furthermore, to be able to include a large number of attributes, the best-worst scaling did not include attribute levels. The importance scores therefore present the patients’ preferences for attributes of DMDs in general; preferences for a specific DMD with a certain attribute level may deviate from the scores obtained in this study. Additionally, although the recruitment method enabled the inclusion of RRMS and CIS patients from all regions of the Netherlands, the samples for the focus groups and the best-worst scaling contained a relatively high proportion of women, older participants and more highly educated participants in comparison with the average MS patient population in the Netherlands [37] and may contain patients who are more involved in their disease management due to our recruitment method via websites and mailing lists of patient organizations. Patients may therefore have a better understanding of what the benefits and risks entail and this could influence the generalizability of the results. Differences were found between men and women in importance of attributes, but subgroup analyses revealed no major difference according to age and level of education of the patient. Moreover, our recruitment method did not enable us to collect data from medical records, and therefore diagnosis, disease duration, relapses experienced and DMD history were self-reported by patients. This may have resulted in incorrect reporting of medical and sociodemographic characteristics. In addition, the transferability of our findings to other settings could be uncertain since the health care system and practice could potentially impact the importance of some attributes, For example, we could expect that the total DMD costs would be more important in countries where patients have out-of-pocket contributions. Lastly, the subgroup analyses had an exploratory purpose. We did not aim to recruit a minimum number of patients for each subgroup. Therefore, the difference in importance of attributes according to the subgroups should be interpreted with caution.

In conclusion, this study shows that patients with RRMS and CIS find beneficial and unwanted effects to be the most important DMD attributes in making decisions about DMD treatment, more important than usability issues with taking the DMD. The effect on disease progression and quality of life were the most important attributes. However, this study also recognizes the heterogeneity in preferences of patients. When having to make a decision about DMD treatment, clinicians should be aware of what the average patient finds important and incorporate information on these attributes in the education for the shared decision making process. However, person-centered clinical decision making requires eliciting the individual patient’s preferences for DMD treatment at the point of the decision.

## Supporting Information

S1 Data Design best-worst scaling. (CSV)

S2 Data Results best-worst scaling. (XLSX)

S1 Fig Results of the best-worst scaling, subgroup analyses. (DOCX)

S1 Table Results of the best-worst scaling, DMD experience. (DOCX)

S1 Text Exploratory phase literature review. (DOCX)

S2 Text Exploratory phase interviews with healthcare professionals. (DOCX)

S3 Text Focus groups with nominal group technique. (DOCX)
