# Supplementary material for: Identification and Prioritization of Important Attributes of Disease-Modifying Drugs in Decision Making among Patients with Multiple Sclerosis: A Nominal Group Technique and Best-Worst Scaling
Source: PLoS One. 2016 Nov 3;11(11):e0164862. doi: 10.1371/journal.pone.0164862 (PMC5094791; doi:10.1371/journal.pone.0164862)
Supplement: S1 Fig — (DOCX) [file pone.0164862.s003.docx]

# S1 Fig Results of the subgroup analyses of the best-worst scaling: difference in the relative importance scores and their significance

Identification and prioritization of important attributes of disease-modifying drugs in decision making among patients with multiple sclerosis: a nominal group technique and best-worst scaling

PLOS ONE

Kremer IEH^*^, Evers SMAA, Jongen PJ, van der Weijden T, van de Kolk I, Hiligsmann M

^*^Corresponding author:

E-mail address: [i.kremer@maastrichtuniversity.nl](mailto:i.kremer@maastrichtuniversity.nl)

# S1 Fig Results of the subgroup analyses of the best-worst scaling

Results of the additional subgroup analyses are presented in fig 1 to 8.


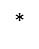

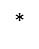

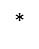

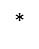

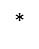

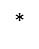


**Fig 1. Attributes' relative importance scores with 95% confidence interval: male (n=25) vs. female (n=160), ordered according to the ranking of the overall analysis.**

DMD, Disease-modifying drugs; MS, multiple sclerosis.

* p< .05, ** p< .0019

Fig 2. Attributes' relative importance scores with 95% confidence interval: age ≤42 (n=93) vs. > 42 (n=92), ordered according to the ranking of the overall analysis.

DMD, Disease-modifying drugs; MS, multiple sclerosis.

* p< .05, ** p< .0019

Fig 3. Attributes' relative importance scores with 95% confidence interval: high (n=113) vs. low education (n=72), ordered according to the ranking of the overall analysis.

DMD, Disease-modifying drugs; MS, multiple sclerosis.

* p< .05, ** p< .0019

Fig 4. Attributes' relative importance scores with 95% confidence interval: disease duration ≤ 4.6 years (n=91) vs. > 4.6 years (n=94), ordered according to the ranking of the overall analysis.

DMD, Disease-modifying drugs; MS, multiple sclerosis.

* p< .05, ** p< .0019

Fig 5. Attributes' relative importance scores with 95% confidence interval: respondents currently taking noDMD (n=54) vs. respondents currently taking a DMD (n=131), ordered according to the ranking of the overall analysis.

DMD, Disease-modifying drugs; MS, multiple sclerosis.

* p< .05, ** p< .0019

Fig 6. Attributes' relative importance scores with 95% confidence interval: respondents currently taking an oralDMD(n=59) vs. respondents currently taking a parenteral (intramuscular, subcutaneous or intravenous administration) DMD (n=72), ordered according to the ranking of the overall analysis.

DMD, Disease-modifying drugs; MS, multiple sclerosis.

* p< .05, ** p< .0019

Fig 7. Attributes' relative importance scores with 95% confidence interval: respondents who did not experience relapses during the last 12 months (n=63) vs. respondents who experienced 1 or more relapses during the last 12 months (n=109), ordered according to the ranking of the overall analysis.

DMD, Disease-modifying drugs; MS, multiple sclerosis.

* p< .05, ** p< .0019

Fig 8. Attributes' relative importance scores with 95% confidence interval: respondents who have not used DMD previously (n=98) vs. respondents who have used 1 or more DMDs previously (n=86), ordered according to the ranking of the overall analysis.

DMD, Disease-modifying drugs; MS, multiple sclerosis.

* p< .05, ** p< .0019
