# Supplementary material for: Identification and Prioritization of Important Attributes of Disease-Modifying Drugs in Decision Making among Patients with Multiple Sclerosis: A Nominal Group Technique and Best-Worst Scaling
Source: PLoS One. 2016 Nov 3;11(11):e0164862. doi: 10.1371/journal.pone.0164862 (PMC5094791; doi:10.1371/journal.pone.0164862)
Supplement: S1 Table — (DOCX) [file pone.0164862.s004.docx]

# S1 Table Results of the subgroup analyses of the best-worst scaling: test statistics of the relative importance scores of DMD naive vs. DMD experienced patients

Identification and prioritization of important attributes of disease-modifying drugs in decision making among patients with multiple sclerosis: a nominal group technique and best-worst scaling

PLOS ONE

Kremer IEH^*^, Evers SMAA, Jongen PJ, van der Weijden T, van de Kolk I, Hiligsmann M

^*^Corresponding author:

E-mail address: [i.kremer@maastrichtuniversity.nl](mailto:i.kremer@maastrichtuniversity.nl)

# S1 Table Results of the subgroup analyses of the best-worst scaling: test statistics of the relative importance scores of DMD naive vs. DMD experienced patients

**Table 1. Results of subgroup analyses DMD naive vs. DMD experienced patients**

| **Attribute** | **DMD naive N=27** | | **DMD experienced N=157** | | **Test statistic: difference** | **p-value** |
| --- | --- | --- | --- | --- | --- | --- |
|  | **Mean RIS (95% CI)** | **Mean rank** | **Mean RIS (95% CI)** | **Mean rank** |  |  |
| **Effect on disease progression** | 9.54  (9.18-9.91) | 81.44 | 9.68  (9.49-9.86) | 94.40 | 1821 (-1,17)^2^ | .244 |
| **Effect on quality of life** | 9.17  (8.41-9.94) | 97.56 | 9.22  (9.01-9.43) | 91.63 | 1983 (-0,53)^2^ | .596 |
| **Effect on relapse rate** | 5.76  (4.54-6.98) | 58.30 | 8.13  (7.76-8.49) | 98.38 | 1196 (-3,61)^2^ | <.001 |
| **Severity of side effects** | 9.30  (8.72-9.88) | 139.04 | 7.33  (7.01-7.66) | 84.50 | 863 (-4,92)^2^ | <.001 |
| **Effect on the severity of relapses** | 5.79  (4.67-6.92) | 62.59 | 7.67  (7.34-8.01) | 97.64 | 1312 (-3,16) ^2^ | .001 |
| **Effect on current MS symptoms** | 6.90  (6.03-7.77) | 81.11 | 7.40  (7.10-7.71) | 94.46 | 1812 (-1,20) ^2^ | .230 |
| **Effect on plaque development in the brain** | 6.49  (5.49-7.49) | 72.70 | 7.47  (7.08-7.87) | 95.90 | 1585 (-2,09) ^2^ | .038 |
| **Safety** | 6.31  (5.05-7.57) | NA | 6.00  (5.54-6.46) | NA | .51^1^ | .611 |
| **Influence on life style** | 5.30  (4.02-6.58) | 92.41 | 5.31  (4.86-5.77) | 92.52 | 2117 (-0,01) ^2^ | .992 |
| **Type of side effects** | 6.97  (6.06-7.89) | NA | 4.63  (4.63-4.22) | NA | 4.36^1^ | <.001 |
| **Effect on life expectancy** | 3.60  (2.49-4.71) | 71.37 | 5.04  (4.54-5.53) | 96.13 | 1549 (-2,23) ^2^ | .026 |
| **Uncertainty about long-term consequences** | 5.34  (4.09-6.60) | 105.67 | 4.42  (4.00-4.84) | 90.24 | 1764 (-1,39) ^2^ | .168 |
| **Duration of side effects** | 5.30  (4.62-5.98) | 135.96 | 3.45  (3.15-3.74) | 85.03 | 946 (-4,59) ^2^ | <.001 |
| **Pace of effect** | 2.55  (1.78-3.31) | 76.89 | 3.27  (2.92-3.62) | 95.18 | 1698 (-1,65) ^2^ | .101 |
| **Insurance coverage** | 2.43  (1.39-3.47) | 89.89 | 2.72  (2.27-3.18) | 92.95 | 2049 (-0,28) ^2^ | .781 |
| **Interaction with other medication** | 1.45  (0.79-2.10) | 84.33 | 1.78  (1.48-2.08) | 93.90 | 1899 (-0,86) ^2^ | .388 |
| **Method of administration** | 1.84  (0.76-2.92) | 106.89 | 1.54  (1.11-1.96) | 90.03 | 1731 (-1,52) ^2^ | .131 |
| **Mode of action of DMD** | 0.97  (0.42-1.52) | 87.33 | 1.00  (0.81-1.19) | 93.39 | 1980 (-0,55) ^2^ | .587 |
| **Further development of DMD** | 1.08  (0.54-1.62) | 99.04 | 0.84  (0.70-0.97) | 91.38 | 1943 (-0,69) ^2^ | .492 |
| **Total DMD costs** | 0.84  (0.35-1.32) | 87.41 | 0.86  (0.66-1.06) | 93.38 | 1982 (-0,54) ^2^ | .593 |
| **Frequency of administration** | 0.67  (0.31-1.04) | 111.70 | 0.68  (0.45-0.91) | 89.20 | 1601 (-2,03) ^2^ | .045 |
| **Required monitoring** | 0.97  (0.34-1.61) | 99.81 | 0.49  (0.31-0.66) | 91.2 | 1922 (-0,77) ^2^ | .441 |
| **Use of DMD among other MS patients** | 0.41  (0.22-0.60) | 107.74 | 0.33  (0.24-0.42) | 89.88 | 1708 (-1,61) ^2^ | .109 |
| **Ease of travelling** | 0.47  (0.07-0.87) | 104.67 | 0.26  (0.13-0.40) | 90.41 | 1791 (-1,29) ^2^ | .200 |
| **Duration of administration** | 0.26  (0.15-0.38) | 110.04 | 0.19  (0.15-0.23) | 89.48 | 1646 (-1,85) ^2^ | .066 |
| **Composition of DMD** | 0.20  (0.11-0.30) | 99.63 | 0.18  (0.12-0.24) | 91.27 | 1927 (-0,75) ^2^ | .460 |
| **Contact person at pharmaceutical company** | 0.08  (0.04-0.11) | 98.02 | 0.11  (0.06-0.15) | 91.55 | 1970,5 (-0,58)^2^ | .566 |

CI, confidence interval; DMD, disease-modifying drug; MS, multiple sclerosis; NA, not applicable; RIS, relative importance score.

^1^ t-statistic (df=182)

^2^ Mann-Whitney U (z-score)
