# Supplementary material for: Identification and Prioritization of Important Attributes of Disease-Modifying Drugs in Decision Making among Patients with Multiple Sclerosis: A Nominal Group Technique and Best-Worst Scaling
Source: PLoS One. 2016 Nov 3;11(11):e0164862. doi: 10.1371/journal.pone.0164862 (PMC5094791; doi:10.1371/journal.pone.0164862)
Supplement: S1 Text — (DOCX) [file pone.0164862.s005.docx]

**S1 Text Exploratory phase: literature review**

Identification and prioritization of important attributes of disease-modifying drugs in decision making among patients with multiple sclerosis: a nominal group technique and best-worst scaling

PLOS ONE

Kremer IEH^*^, Evers SMAA, Jongen PJ, van der Weijden T, van de Kolk I, Hiligsmann M

^*^Corresponding author:

E-mail address: [i.kremer@maastrichtuniversity.nl](mailto:i.kremer@maastrichtuniversity.nl)

**S1 Text Exploratory phase: literature review**

# Methods

A literature search was conducted to identify any articles reporting on attributes of DMDs that are of importance for decision making in DMD treatment from a patient’s or healthcare provider’s perspective. Any articles (quantitative and qualitative research, expert opinions) or abstracts were included in this review that reported on DMD attributes for decision making from a RRMS or CIS patient’s or healthcare provider’s perspective. Three search strings were entered in PubMed, EMBASE and PsycINFO, and were searched from November 2004 until November 2014. The search was restricted to articles reported in English, Dutch, German and French. Figure 1 provides the search strings for PubMed. Additionally, abstracts from conferences on MS (CMSC ACTRIMS Annual Meetings, Joint ACTRIMS-ECTRIMS meeting) and shared decision making (Society for Medical Decision Making, International Shared Decision Making Conference) were searched up to 1 year (November 2013 until November 2014). Selection of articles was conducted by consecutively assessing the relevance according to title, abstract and full text. Reference lists of identified articles were searched for any additional articles that match the inclusion criteria. DMD attributes that were reported as being of importance for patients, neurologists and/or MS nurses in the decision making process about DMD treatment for MS were combined into a list with a description of the attribute, if provided. The list was completed with attributes included in DMD decision aids for MS known to the authors and studies on reasons for DMD non-adherence in MS patients.

# Results

The electronic search and reference check resulted in 1,213 unique articles. Selection of relevant articles on preferences for DMD attributes based on title and/or abstract resulted in the exclusion of 1,143 articles. Of the 70 remaining articles, 55 articles were excluded because they did not focus on patients’ or healthcare providers’ preferences for DMD attributes for treatment of RRMS or CIS or because of the study design. Fifteen references were considered relevant for this review. Full articles (n=10) as well as abstracts (n=5) reporting important DMD attributes for decision making were included. The results of the selection procedure are presented in figure 2. Of the 15 studies, 2 were qualitative [[1](#_ENREF_1), [2](#_ENREF_2)] and were 2 expert opinions [[3](#_ENREF_3), [4](#_ENREF_4)]. Out of the quantitative studies, 6 were conjoint analysis [[5-10](#_ENREF_5)] and 5 were surveys [[11-15](#_ENREF_11)]. Most articles reported attributes from the patients’ perspective. Only 3 articles were found about the healthcare providers’ perspective [[3](#_ENREF_3), [4](#_ENREF_4), [13](#_ENREF_13)].

In total, 19 attributes were found. Three main categories of attributes could be identified, each consisting of multiple attributes: attributes related to benefits of DMDs, attributes related to unwanted effects of DMDs, and attributes related to ease of use of DMDs. A fourth category “others” consisted of treatment adherence, composition of DMDs and cost. The attributes derived from the 15 articles were compared with attributes from existing MS decision aids [[16-18](#_ENREF_16)] and a selection of studies focusing on patients’ reasons for non-adherence [[19-21](#_ENREF_19)] to evaluate whether major attributes were missing. One attribute, “impact on daily life”, was added. Table 1 presents all 20 attributes and a summary of any provided descriptions in the literature.

Fig 1. Search strategy in PubMed

*Search string 1.*

multiple sclerosis [MeSH] OR multiple sclerosis, relapsing-remitting [MeSH] OR multiple

sclerosis[tiab] OR ms[tiab] OR clinically isolated syndrome[tiab] OR cis[tiab]

AND

patient preference[MeSH] OR preference[tiab] OR preferences[tiab] OR perspective[tiab]OR perspectives[tiab] OR desire[tiab]OR desires[tiab]OR view[tiab]OR views[tiab]viewpoint[tiab]OR attitude[tiab] OR attitudes[tiab] OR decision[tiab]OR decisions[tiab]patient value[tiab] OR patient value[tiab] OR patients values[tiab] OR patients values[tiab]OR physician value[tiab] OR physician values[tiab]OR physicians value[tiab] OR physicians values[tiab]OR nurse value[tiab] OR nurse values[tiab]OR nurses value[tiab] OR nurses values[tiab]

AND

Immunosuppressive agents[MeSH] OR immunotherapy[MeSH] OR disease modif*[tw] OR immunosuppress*[tw] OR immunomodulat*[tw] OR immunotherap*[tw]

*Search string 2.*

multiple sclerosis [Mesh] OR multiple sclerosis, relapsing-remitting [Mesh] OR multiple

sclerosis[tiab] OR ms[tiab] OR relapsing remitting multiple sclerosis[tiab] OR remitting-relapsing multiple sclerosis[tiab] OR Clinically isolated syndrome[tiab] OR cis[tiab]

AND

Immunosuppressive agents[MeSH] OR immunotherapy[MeSH] OR disease modif*[tw] OR immunosuppress*[tw] OR immunomodulat*[tw] OR immunotherap*[tw]

AND

decision support techniques [MeSH] OR decision making, computer-assisted [MeSH] OR decision support*[tiab] OR decision aid*[tiab] OR decision tool*[tiab] OR decision instrument*[tiab] OR decision technolog*[tiab] OR decision technique*[tiab] OR decision system*[tiab] OR decision program*[tiab] OR decision algorithm*[tiab] OR decision method*[tiab] OR decision intervention*[tiab] OR decision material [tiab]

*Search string 3.*

multiple sclerosis [Mesh] OR multiple sclerosis, relapsing-remitting [Mesh] OR multiple

sclerosis[tiab] OR ms[tiab] OR relapsing remitting multiple sclerosis[tiab] OR remitting-relapsing multiple sclerosis[tiab] OR Clinically isolated syndrome[tiab] OR cis[tiab]

AND

Immunosuppressive agents[MeSH] OR immunotherapy[MeSH] OR disease modif*[tw]OR immunosuppress*[tw] OR immunomodulat*[tw] OR immunotherap*[tw]

AND

interview*[tiab] OR interviews[MeSH:noexp] OR experience*[Tw] OR qualitative[tiab]

Fig 2. Flow chart literature review

Electronic database search

- 754 through PubMed
- 765 through EMBASE
- 128 through PsycINFO

Other sources

- 2 through reference check

1213 unique articles screened on title/ abstract

70 articles screened on full text (if available)

15 articles included

Excluded through title/ abstract screening

220 not fulfilling population

922 not fulfilling DMD attribute preferences

1 not fulfilling study design (protocol)

Excluded through full text screening

1 not fulfilling population

47 not fulfilling DMD attribute preferences

7 not fulfilling study design (protocol)

DMD, disease-modifying drugs.

Table 1. Attributes and their descriptions derived from literature review

| **Attribute** | **Description** | **Articles** |
| --- | --- | --- |
| Attributes related to benefits | | |
| Effect  (in general) | Efficacy, clinical benefits, success rate, burden of disease or treatment failure | Bhanegaonkar 2011;  Gasperini 2011; Goodin 2007;  Hanson 2013; Hanson 2014; Sommers 2009; Visser 2011 |
| Effect on relapse frequency |  | Goodin 2007; Hanson 2014; Johnson 2009; Sommers 2009; Wilson 2014a; Wilson 2014b |
| Effect on worsening of lesions in the brain | Prevention of formations of new or enhancing T2 lesions in the brain | Goodin 2007; Hanson 2014; Wilson 2014a; Wilson 2014b |
| Effect on disability progression | Worsening of disability or MS symptoms (EDSS score) without relapses | Goodin 2007; Hanson 2014; Johnson 2009; Wilson 2014b |
| Effect on MS symptoms | Feeling better due to improvements in existing MS symptoms | Wilson 2014a; Wilson 2014b |
| Pace of effect |  | Visser 2011 |
| Action of DMD | Uncertainty as to the action of the DMD | Visser 2011 |
| Attributes related to unwanted effects | | |
| Side effects | Articles reporting on side effects or adverse events differed greatly in how side effects were classified, according to the type and/or severity of side effects. These side effects are considered to be minor but uncomfortable or common side effects.  E.g. local or systemic, tolerability, mild-significant-severe risk and vigilance, injection site reactions, flu-like symptoms, gastro-intestinal symptoms, lipoatrophy, depression, vision changes, headaches, muscle joint ache, infections | Bergmann 2014; Bhanegaonkar 2011; Coan 2011; Gasperini 2011; Goodin 2007; Gustavsson 2011; Hanson 2013; Hanson 2014; Miller 2006; Utz 2014; Visser 2011; Wilson 2014a; Wilson 2104b |
| (long-term) Safety | Risk on life-threatening or severely disabling side effects, such as liver failure, PML, or leukaemia, also after the patient has stopped using the DMD | Gasperini 2011; Goodin 2007; Hanson 2013;  Hanson 2014; Johnson 2009; Miller 2006; Visser 2011 |
| Time on market | Time duration a DMD has been used | Wilson 2014b |
| Attributes related to ease of use of DMD | | |
| Convenience  (in general) | Convenience in administration of the DMD | Bhaengoankar 2011; Gasperini 2011; Hanson 2013; Hanson 2014 |
| Mode of administration | Pill, intramuscular injection, subcutaneous injection or intravenous infusion. | Bergmann 2014; Gustavsson 2011; Hanson 2013; Miller 2006; Sommers 2009; Utz 2014; Visser 2011; Wilson 2014a; Wilson 2014b |
| Frequency of administration | Frequency of administration per day, week or month | Bergmann 2014; Gustavsson 2011; Utz 2014; Visser 2011; Wilson 2014a; Wilson 2014b |
| Duration of administration |  | Gustavsson 2011 |
| Required monitoring |  | Bergmann 2014; Gasperini 2011; Gustavsson 2011 |
| Impact of DMD on daily life | Consisting of the frequency of administrating the DMD; the method of storing the DMD and its consequences for traveling; required preparation before administration; and whether the DMD contains animal or human products. | University College London NHS Trust 2004 |
| Others | | |
| Composition of DMD | Natural or chemical ingredients in the DMD | Miller 2006 |
| Treatment adherence |  | Bhanegaonkar 2011; Gasperini 2011; Goodin 2007 |
| Cost/ cost-effectiveness | Healthcare coverage or out-of-pocket expenses | Bhanegaonkar 2011; Gasperini 2011; Hanson 2013; Miller 2006; Sommers 2009 |

DMD, disease-modifying drug.

**References**

# References

1. Bhanegaonkar A, Madhavan S, Pawar GV, Rajagopalan K, Langlois J. Understanding health related quality of life changes and issues related to disease modifying drugs among multiple sclerosis patients: a qualitative study. Value Health. 2011;14 (3):A211..

2. Miller CE, Jezewski MA. Relapsing MS patients' experiences with glatiramer acetate treatment: a phenomenological study. J Neurosci Nurs. 2006;38(1):37-41..

3. Gasperini C, Ruggieri S. Emerging oral drugs for relapsing-remitting multiple sclerosis. Expert Opin Emerg Drugs. 2011;16(4):697-712. doi: 10.1517/14728214.2011.642861.

4. Goodin DS, Biermann LD, Bohlega S, Boiko A, Chofflon M, Gebeily S, et al. Integrating an evidence-based assessment of benefit and risk in disease-modifying treatment of multiple sclerosis. Curr Med Res Opin. 2007;23(11):2823-32. doi: 10.1185/03007x233007.

5. Bergmann A, Lang M, Bischoff CL, Schicklmaier P, Nolting HD, Schiffhorst G, et al. Patient preferences in the choice of disease modifying drugs for multiple sclerosis. Neurology. 2014;1.

6. Gustavsson A, Karampampa K, Miltenburger C, Eckert B. Treatment experience, burden, and unmet needs (TRIBUNE) in multiple sclerosis study: patient preferences for MS treatments. Multi Scler. 2011;1:S239. doi: http://dx.doi.org/10.1177/1352458511422300.

7. Johnson FR, Van Houtven G, Özdemir S, Hass S, White J, Francis G, et al. Multiple Sclerosis patients' benefit-risk preferences: serious adverse event risks versus treatment efficacy. J Neurol. 2009;256(4):554-62. doi: 10.1007/s00415-009-0084-2.

8. Utz KS, Hoog J, Wentrup A, Berg S, Lammer A, Jainsch B, et al. Patient preferences for disease-modifying drugs in multiple sclerosis therapy: a choice-based conjoint analysis. Ther Adv Neurol Disord. 2014;7(6):263-75. doi: 10.1177/1756285614555335.

9. Wilson L, Loucks A, Bui C, Gipson G, Zhong L, Schwartzburg A, et al. Patient centered decision making: use of conjoint analysis to determine risk-benefit trade-offs for preference sensitive treatment choices. J Neurol Sci. 2014;344(1-2):80-7. doi: 10.1016/j.jns.2014.06.030.

10. Wilson LS, Loucks A, Gipson G, Zhong L, Bui C, Miller E, et al. Patient preferences for attributes of disease-modifying multiple sclerosis therapies: development and results of a ratings-based conjoint analysis. Int J MS Care. 2015. doi: 10.7224/1537-2073.2013-053.

11. Caon C, Memon A, Perumal J, Khan O. Patient response to new disease-modifying therapies: results of a questionnaire study in RRMS patients receiving selfinjected disease-modifying therapies. Multiple Sclerosis. 2011;1:S472. doi: http://dx.doi.org/10.1177/1352458511422301.

12. Hanson KA, Agashivala N, Stringer SM, Balantac Z, Brandes DW. A cross-sectional survey of patient satisfaction and subjective experiences of treatment with fingolimod. Patient Prefer Adherence. 2013;7:309-18. doi: 10.2147/ppa.s41992.

13. Hanson KA, Agashivala N, Wyrwich KW, Raimundo K, Kim E, Brandes DW. Treatment selection and experience in multiple sclerosis: survey of neurologists. Patient Prefer Adherence. 2014;8:415-22. Epub 2014/04/15. doi: 10.2147/ppa.s53140.

14. Sommers R, Phillips A. Unmet needs in the treatment of multiple sclerosis: patient perspectives. Journal of Managed Care Pharmacy. 2009;15 (7):602-3.

15. Visser LH, van der Zande A. Reasons patients give to use or not to use immunomodulating agents for multiple sclerosis. Eur J Neurol. 2011;18(11):1343-9. doi: 10.1111/j.1468-1331.2011.03411.x.

16. Kasper J, Kopke S, Muhlhauser I, Nubling M, Heesen C. Informed shared decision making about immunotherapy for patients with multiple sclerosis (ISDIMS): a randomized controlled trial. Eur J Neurol. 2008;15(12):1345-52. doi: 10.1111/j.1468-1331.2008.02313.x.

17. University College London NHS Trust. MS Decisions: an independent aid to your decision 2004 (revised in 2009). Available from: www.msdecisions.org.uk.

18. Winn K, Oliver BJ. Shared decision making in multiple sclerosis: option grid point of care engagement tools for disease-modifying treatment (work in progress) Int J MS Care. 2014;16(Suppl 3):S20.

19. Bischoff C, Schreiber H, Bergmann A. Background information on multiple sclerosis patients stopping ongoing immunomodulatory therapy: a multicenter study in a community-based environment. J Neurol. 2012;259(11):2347-53. doi: 10.1007/s00415-012-6499-1..

20. Fox RJ, Salter AR, Tyry T, Sun J, You X, Laforet G, et al. Treatment discontinuation and disease progression with injectable disease-modifying therapies: findings from the north american research committee on multiple sclerosis database. Int J MS Care. 2013;15(4):194-201. doi: 10.7224/1537-2073.2012-034.

21. Salter AR, Marrie RA, Agashivala N, Belletti DA, Kim E, Cutter GR, et al. Patient perspectives on switching disease-modifying therapies in the NARCOMS registry. Patient Prefer Adherence. 2014;8:971-9. doi: 10.2147/ppa.s49903.
