# Supplementary material for: Identification and Prioritization of Important Attributes of Disease-Modifying Drugs in Decision Making among Patients with Multiple Sclerosis: A Nominal Group Technique and Best-Worst Scaling
Source: PLoS One. 2016 Nov 3;11(11):e0164862. doi: 10.1371/journal.pone.0164862 (PMC5094791; doi:10.1371/journal.pone.0164862)
Supplement: S2 Text — (DOCX) [file pone.0164862.s006.docx]

**S2 Text Exploratory phase: interviews with healthcare professionals**

Identification and prioritization of important attributes of disease-modifying drugs in decision making among patients with multiple sclerosis: a nominal group technique and best-worst scaling

PLOS ONE

Kremer IEH^*^, Evers SMAA, Jongen PJ, van der Weijden T, van de Kolk I, Hiligsmann M

^*^Corresponding author:

E-mail address: [i.kremer@maastrichtuniversity.nl](mailto:i.kremer@maastrichtuniversity.nl)

**S2 Text Exploratory phase: interviews with healthcare professionals**

# Methods

Interviews with MS-related healthcare professionals were conducted to obtain their perspectives on important DMD attributes in the decision making about DMD treatment. A convenience sample of 7 neurologists and 7 MS-specialized nurses employed in hospital in the Netherlands were contacted by e-mail (P.J.) to invite them to participate in a 20-minute telephone interview. All contacted healthcare professionals consulted RRMS or CIS patients about DMD treatment on a regular basis. Healthcare professionals that were willing to participate were asked to contact I.K. to schedule the interview. Telephone interviews were fitting for the structured form and not sensitive content of the interview. At the beginning of the interview, demographic characteristics and work experience with MS patients were recorded. Each healthcare provider was asked which DMD attributes they considered to be of importance in the decision making about DMD treatments for patients. After the healthcare provider’s attributes were elicited, his/her perspective on any additional attributes derived from literature was asked. Audio recordings of interviews were used to enable transcription of the attribute descriptions and were deleted afterwards. Each interview resulted in a list of attributes that are of importance according to the healthcare provider. One overall attribute list was created by comparing all attributes from the interviews with the attributes derived from literature.

# Results

Six healthcare professionals (3 neurologists, 3 MS-specialized nurses) were willing and able to participate in the interview. The neurologists all treated 200 or more MS patients yearly and had work experience ranging between 8 and 24 years. Participating nurses had 10 to 14 years of work experiences as an MS-specialized nurse. Further details on characteristics of the health professionals are presented in table 1. Four attributes were reported by the healthcare professionals that were not found in the literature: 1) effect on quality of life for the patient; 2) effect on brain atrophy, 3) ease of use for the neurologist, meaning the degree of complexity for the neurologist regarding authorization from health insurance or procedures regarding delivery of DMD; 4) total costs of DMDs, meaning that the total costs need to be considered as part of the Dutch Healthcare budget if a patient doubts whether to use a DMD. Therefore, costs had a broader definition than costs as elicited from the literature, which represented insurance coverage or out-of-pocket expenses.

Table 1. Characteristics of healthcare professionals

|  | **Neurologists (N=3)** | **MS nurses (N=3)** |
| --- | --- | --- |
| Gender  Male n (%)  Female n (%) | 3 (100)  0 (0) | 0 (0)  100 (0) |
| Age (years)  Mean ± SD | 50.7 ± 8.1 | 52.3 ± 3.1 |
| Work experience (years)  Mean ± SD | 18 ± 8.7 | 12.0 ± 2.0 |

MS, multiple sclerosis; SD, standard deviation.
