# Supplementary material for: Identification and Prioritization of Important Attributes of Disease-Modifying Drugs in Decision Making among Patients with Multiple Sclerosis: A Nominal Group Technique and Best-Worst Scaling
Source: PLoS One. 2016 Nov 3;11(11):e0164862. doi: 10.1371/journal.pone.0164862 (PMC5094791; doi:10.1371/journal.pone.0164862)
Supplement: S3 Text — (DOCX) [file pone.0164862.s007.docx]

**S3 Text Focus groups with nominal group technique**

Identification and prioritization of important attributes of disease-modifying drugs in decision making among patients with multiple sclerosis: a nominal group technique and best-worst scaling

PLOS ONE

Kremer IEH^*^, Evers SMAA, Jongen PJ, van der Weijden T, van de Kolk I, Hiligsmann M

^*^Corresponding author:

E-mail address: [i.kremer@maastrichtuniversity.nl](mailto:i.kremer@maastrichtuniversity.nl)

**S3 Text Focus groups with nominal group technique**

# Results

Table 1 presents all DMD attributes that influence the decision making about DMD treatment as identified by patients in the focus groups. In table 2, the ranking of all reported DMD attributes is presented with the frequency with which they were included in the top 10 and top 5 and their mean importance scores. Compared to the results of the literature review and interviews with healthcare providers, 12 additional attributes were reported. Furthermore, participants described 4 attributes differently from literature and healthcare providers. Side effects were split up into type, duration and severity of non-life threatening side effects. Influence on life style was described by patients as the required changes in habits and activities one has to make in contrast to the literature which incorporates administration frequency, ease of traveling and having a premixed syringe available. Traveling was reported as a separate attribute by the patients and having a premixed syringe was considered to be a part of the method of administration or making traveling easier. Time on the market had a comparable description as uncertainty about long-term consequences and was therefore deleted. Composition of the DMD was of importance for possible allergic reactions instead of the natural or chemical components. Two attributes were found not to be of importance for patients: effect on brain atrophy and ease of use for neurologists.

Table 1. Attributes and their descriptions as derived from the focus groups

| DMD attribute | Description |
| --- | --- |
| Effect on disease progression/ becoming disabled | Proven inhibitory effect of the DMD on developing physical and cognitive disabilities on the long-term. |
| Effect on current MS symptoms | Proven effect of the DMD on reducing MS symptoms, especially fatigue, that someone is experiencing at the moment. |
| Effect on the number of relapses | Proven inhibitory effect of the DMD on the number of relapses experienced by a person. |
| Effect on the severity of relapses | Proven effect of the DMD on reducing the severity of relapses. |
| Effect on development of plaques in the brain | Proven inhibitory effects of the DMD on the development and presence of plaques (scarring) in the brain which can be identified on an MRI. |
| Effect on quality of life | Proven effect on improving the overall (physical, mental and social) well-being of a patient as a result of the DMD [[1](#_ENREF_1)]. |
| Effect on life expectancy | Proven effect of the DMD on prolonging life duration. |
| Pace of effect | Speed at which the beneficial effects occur after administrating the DMD |
| Action of DMD | The way the DMD works in the body and therefore has beneficial effects, e.g. reducing inflammations that cause nerve damage. |
| Safety | Risks of serious side effects that can be life-threatening or result in severe disabilities. |
| Type of side effects | Risk of physical or psychological side effects that are not life-threatening or leading to severe disabilities. Examples of these side effects are injection site reactions, panic attacks, gastrointestinal symptoms, flu-like symptoms, hair loss, and flushing. |
| Duration to which side effects persist | The extent to which effects are temporary (e.g. muscle pain) or permanent (e.g. subcutaneous lumps) |
| Severity of side effects | The extent to which the severity of the side effects outweigh the desire to treat MS. |
| Uncertainty about long-term consequences | The lack of knowledge and experience regarding serious side effects on the long-term, especially for medication that have been only shortly available on the market. |
| Method of administration | The form in which the DMD is to be taken (tablet, injection or infusion), and whether this can be self-administered or whether help is needed. |
| Frequency of administration | How often the DMD should be taken per day, week or month. |
| Duration of administration | Length of time that the administration of one dose takes up. |
| Traveling | The ease with which the DMD can be brought on holidays or outings. The extent to which cooling of the DMD is needed and regulations regarding possession of the medication when traveling abroad is considered. |
| Required monitoring | Medical checks that are required during the use of the DMD, such as blood tests and MRIs. |
| Use of DMD among other MS patients | The proportion of MS patients in the country that is currently using the specific DMD. |
| Insurance coverage | The extent to which the medication is reimbursed by the health insurance. |
| Total costs | Total costs of the DMD for the health insurance, independent of the out-of-pocket expenses for the patient |
| Interaction with other medication | Interaction of the DMD in combination with other medication, including contraception, which may change the effectiveness of either one of the medications or which may result in additional side effects. |
| Composition of DMD | The substances processed in the DMD that may cause allergic reactions. |
| Contact person at pharmaceutical company | The extent to which help from the pharmaceutical company is available, such as a nurse, which the patient can contact about questions regarding side effects and administration. |
| Development of DMD | The extent to which the DMD will remain in constant development after has become available on the market, in order to fine-tune the DMD in effectiveness and side-effects |
| Influence on lifestyle | The extent to which a patient’s habits or lifestyle have to be adjusted for proper use of the medication, such as the extent of flexibility in time of administration, being able and allowed to drive, work, do sports, drink alcohol, etc. |
| Adherence rates | The known rates for a specific DMD of patients persisting to use the DMD over a long period of time, following the prescribed dose. |
| Availability of DMD in the Netherlands | Whether the DMD is available in the Netherlands or whether it must be obtained abroad. |
| Brand recognition | The extent to which the DMD is known to patients because it is appointed by neurologists, nurses, websites and other information sources |
| Legal liability | Whether a statement of legal liability needs to be signed before being able to start the DMD |
| Issuance of DMD | The method of acquiring the DMD, e.g. method of delivery, place it can be picked up. |
| Wash out requirement | Minimum required wash out period and method in case of stopping or switching treatments. |
| Shelf life | Duration the DMD can be kept before using it. |
|  |  |

DMD, disease-modifying drug; MS, multiple sclerosis.

**Table 2.** Ranking and attribute scores derived from focus groups

| Ranking | Attribute | Top 10 | Top 5 | Mean score |
| --- | --- | --- | --- | --- |
| 1 | Type of side effects | 12 | 11 | 2.11 |
| 2 | Effect on disease progression/ becoming disabled | 11 | 9 | 1.82 |
| 3 | Method of administration | 16 | 9 | 1.58 |
| 4 | Effectiveness in reducing relapse rate | 13 | 8 | 1.45 |
| 5 | Safety | 15 | 9 | 1.16 |
| 6 | Insurance coverage | 13 | 8 | 0.95 |
| 7 | Duration to which side effects persist | 5 | 4 | 0.74 |
| 8 | Influence on lifestyle | 12 | 7 | 0.63 |
| 9 | Required monitoring | 8 | 5 | 0.58 |
| 10 | Frequency of administration | 8 | 3 | 0.58 |
| 11 | Effect on current MS symptoms | 7 | 3 | 0.55 |
| 12 | Severity of side effects | 6 | 3 | 0.47 |
| 13 | Traveling | 7 | 4 | 0.32 |
| 14 | Effect on quality of life | 4 | 3 | 0.32 |
| 15 | Uncertainty about long-term consequences | 8 | 2 | 0.32 |
| 16 | Effect on life expectancy | 3 | 2 | 0.32 |
| 17 | Interaction with other medication | 7 | 2 | 0.26 |
| 18 | Action of DMD | 3 | 1 | 0.26 |
| 19 | Pace of effect | 4 | 1 | 0.21 |
| 20 | Effect on development of plaques in the brain | 4 | 2 | 0.13 |
| 21 | Contact person at pharmaceutical company | 2 | 1 | 0.11 |
| 22 | Development of DMD | 6 | 1 | 0.05 |
| 23 | Use of DMD among other MS patients | 3 | 1 | 0.05 |
| 24 | Total costs | 1 | 1 | 0.05 |
| 25 | Duration of administration | 2 | 0 | 0 |
|  | Composition of DMD | 2 | 0 | 0 |
|  | Effect on the severity of relapses | 2 | 0 | 0 |
| 28 | Adherence rates | 0 | 0 | 0 |
|  | Availability of DMD in the Netherlands | 0 | 0 | 0 |
|  | Brand recognition | 0 | 0 | 0 |
|  | Issuance of DMD | 0 | 0 | 0 |
|  | Wash out requirement | 0 | 0 | 0 |
|  | Shelf life | 0 | 0 | 0 |
|  | Legal liability | 0 | 0 | 0 |

DMD, disease-modifying drug; MS, multiple sclerosis.

# Reference

1. Sprangers M. Wat is kwaliteit van leven en hoe wordt het gemeten? Volksgezondheid Toekomst Verkenning, Nationaal Kompas Volksgezondheid. Bilthoven. http://www.nationaalkompas.nl/gezondheid-en-ziekte/functioneren-en-kwaliteit-van-leven/kwaliteit-van-leven/wat-is-kwaliteit-van-leven-en-hoe-wordt-het-gemeten/ Accessed 6 May 2015: RIVM; 2013.
